# Supplementary material for: Antimicrobial resistance determinants are associated with Staphylococcus aureus bacteraemia and adaptation to the healthcare environment: a bacterial genome-wide association study
Source: Microb Genom. 2021 Nov 23;7(11):000700. doi: 10.1099/mgen.0.000700 (PMC8743558; doi:10.1099/mgen.0.000700)
Supplement: Supplementary material 1 [file mgen-7-0700-s001.pdf]

Antimicrobial resistance determinants are associated with *Staphylococcus aureus* bacteraemia and adaptation to the healthcare environment: a bacterial genome-wide association study

Supplemental information

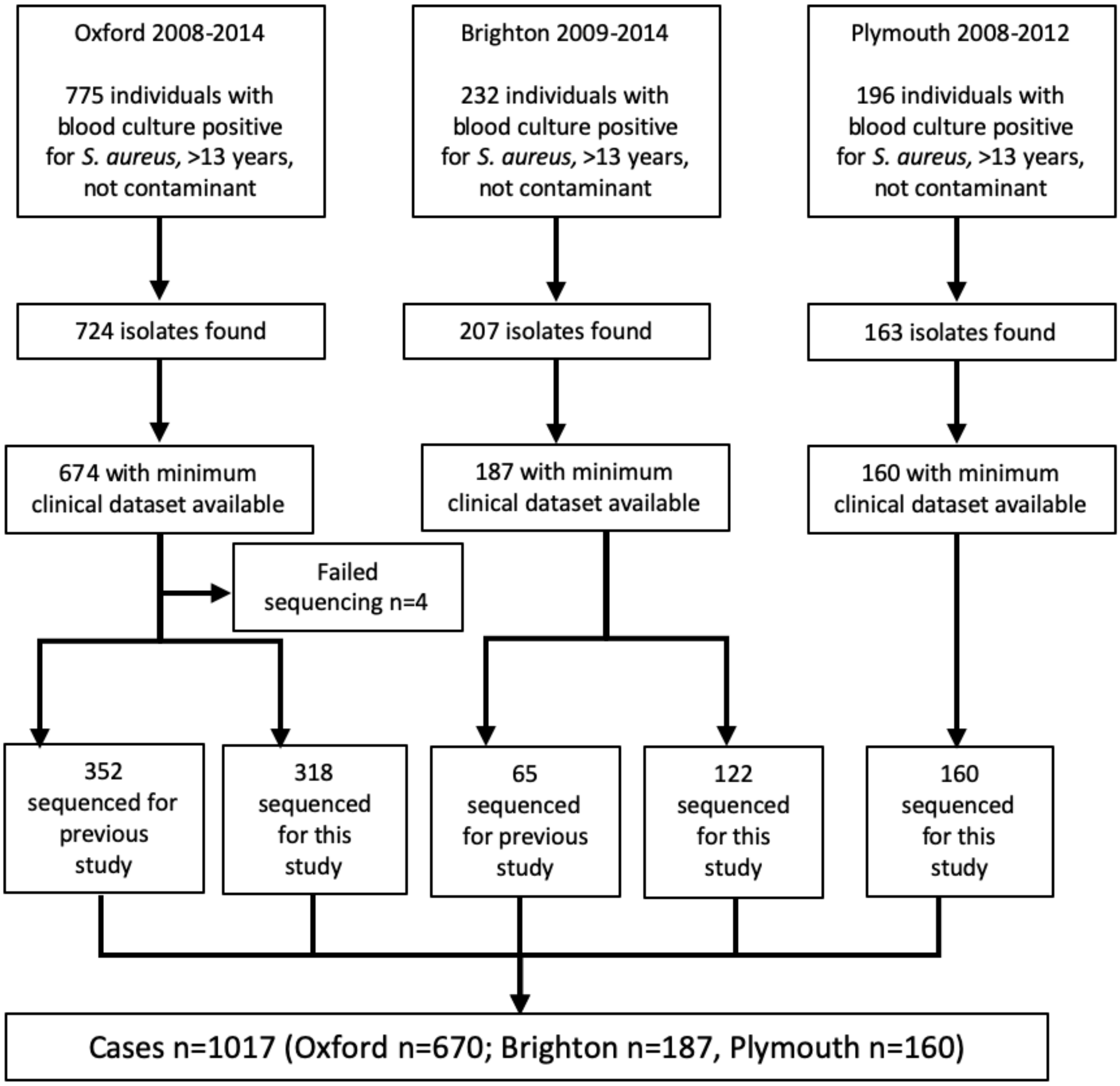

Figure S1: Flow diagram of cases screened for inclusion in the study

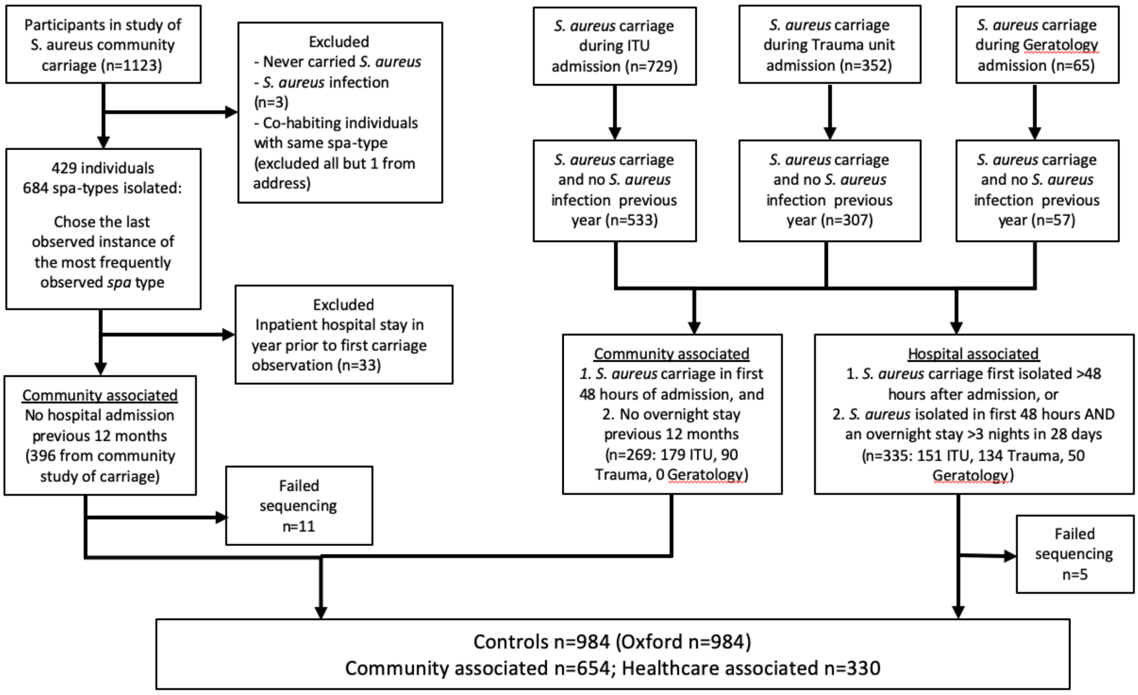

Figure S2: Flow diagram of controls screened for inclusion in the study

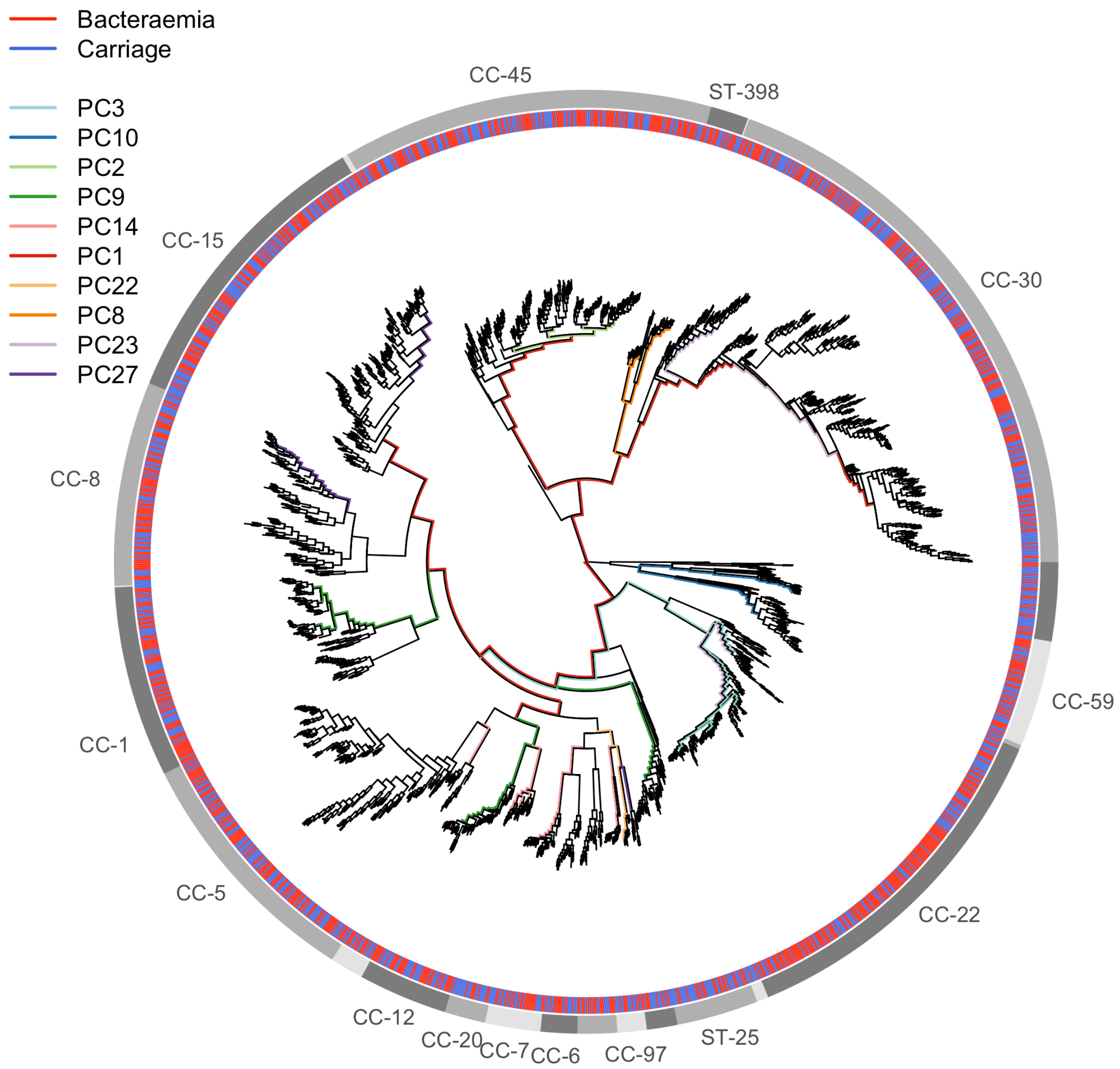

Bacteraemia
Carriage
PC3
PC10
PC2
PC9
PC14
PC1
PC22
PC8
PC23
PC27
CC-45
ST-398
CC-30
CC-15
CC-8
CC-1
CC-59
CC-5
CC-22
CC-12
CC-20
CC-7
CC-6
CC-97
ST-25

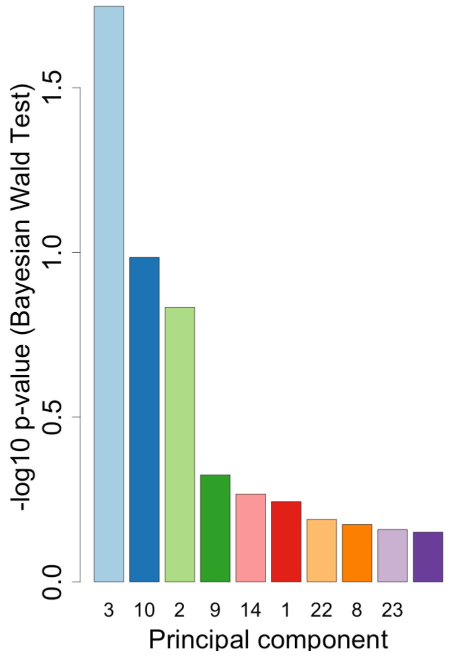

-log10 p-value (Bayesian Wald Test)
0.0
0.5
1.0
1.5
3
10
2
9
14
1
22
8
23
Principal component

Figure S3: Association of Principal Components with bacteraemia-vs-carriage. (A) The branches corresponding to the 10 lineages most significantly associated with bacteraemia-vs-carriage are coloured on a maximum likelihood phylogeny of the study isolates. Branch lengths have been square-root transformed to discriminate closely related lineages. Clonal complexes are denoted in the outermost ring. The second outermost ring indicates isolate source (blue carriage, red bacteraemia) **(B)** Significance (negative $\log_{10}$ *p*-values) of the 10 most significantly associated PCs. A Bonferroni corrected threshold for significance is $10^{-4.6}$.

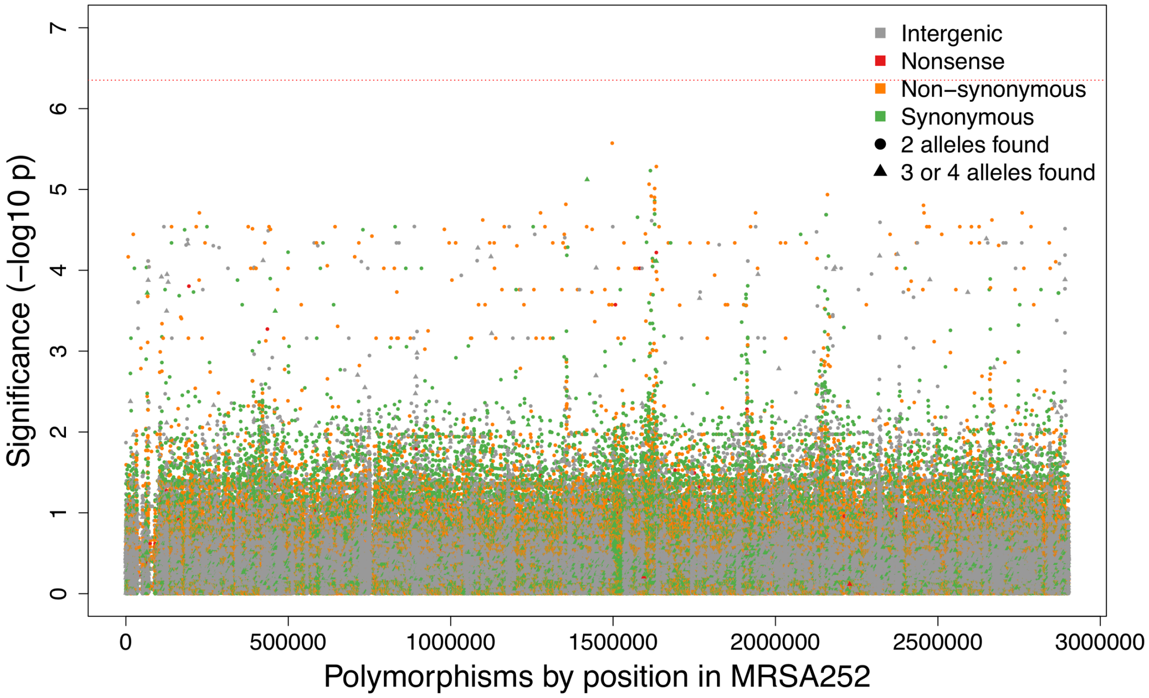

Figure S4: Manhattan plot of SNP associations with bacteraemia-vs-carriage, controlling for population structure and HA or CA origin. The significance of each SNP ($-\log_{10} p$ value LRT) is plotted according to location on the 2.9MB MRSA252 reference genome, with control for population structure and HA-vs-CA origin. SNPs are coloured according to their predicted effect on protein, and shaped according to the number of alleles found at that site in the study set. A Bonferroni-corrected significance threshold based on the number of phylopatterns (n=112, 292) is plotted in red ($10^{-6.4}$), assuming a family-wise error rate of 5%.

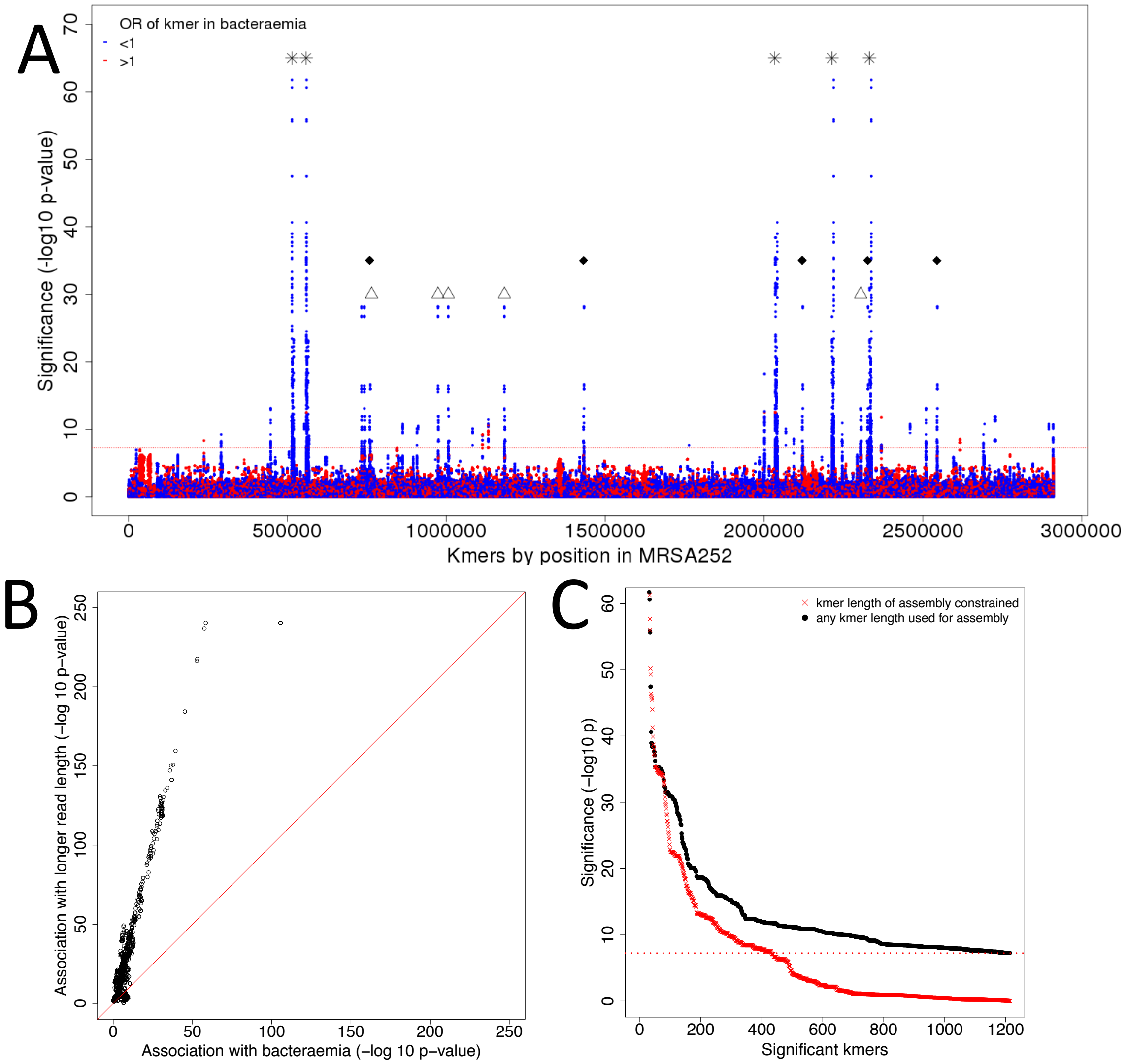

Figure S5: Confounding of kmer study by sequencing read length. (A) Manhattan plot of kmers associated with phenotype in study of 2001 isolates. Kmers are mapped to reference genome and plotted against statistical significance of association with case-control-status. Kmers found more frequently in carriage are mapped in blue, kmers found more frequently in bacteraemia are mapped in red. A Bonferroni corrected threshold for significance is plotted in red ($10^{-7.3}$). The positions of several repeat regions are marked: 16S rDNA (*), ISS (Δ) and ISSSau3 (♦). **(B)**The kmers most associated with bacteraemia-vs-carriage were confounded with sequence length. We plotted significance of association with both bacteraemia-vs-carriage (X axis) and sequencing read length (Y axis), using a χ^2 test, without correction for population structure. **(C)** Kmer detection and association testing with GEMMA was repeated, this time on new assemblies, built using Velvet with a maximum kmer length of 79bp. The signal of the most significant kmers based on the original assemblies (black) dropped to below statistical significance when the assemblies were built with a constrained kmer length (red).

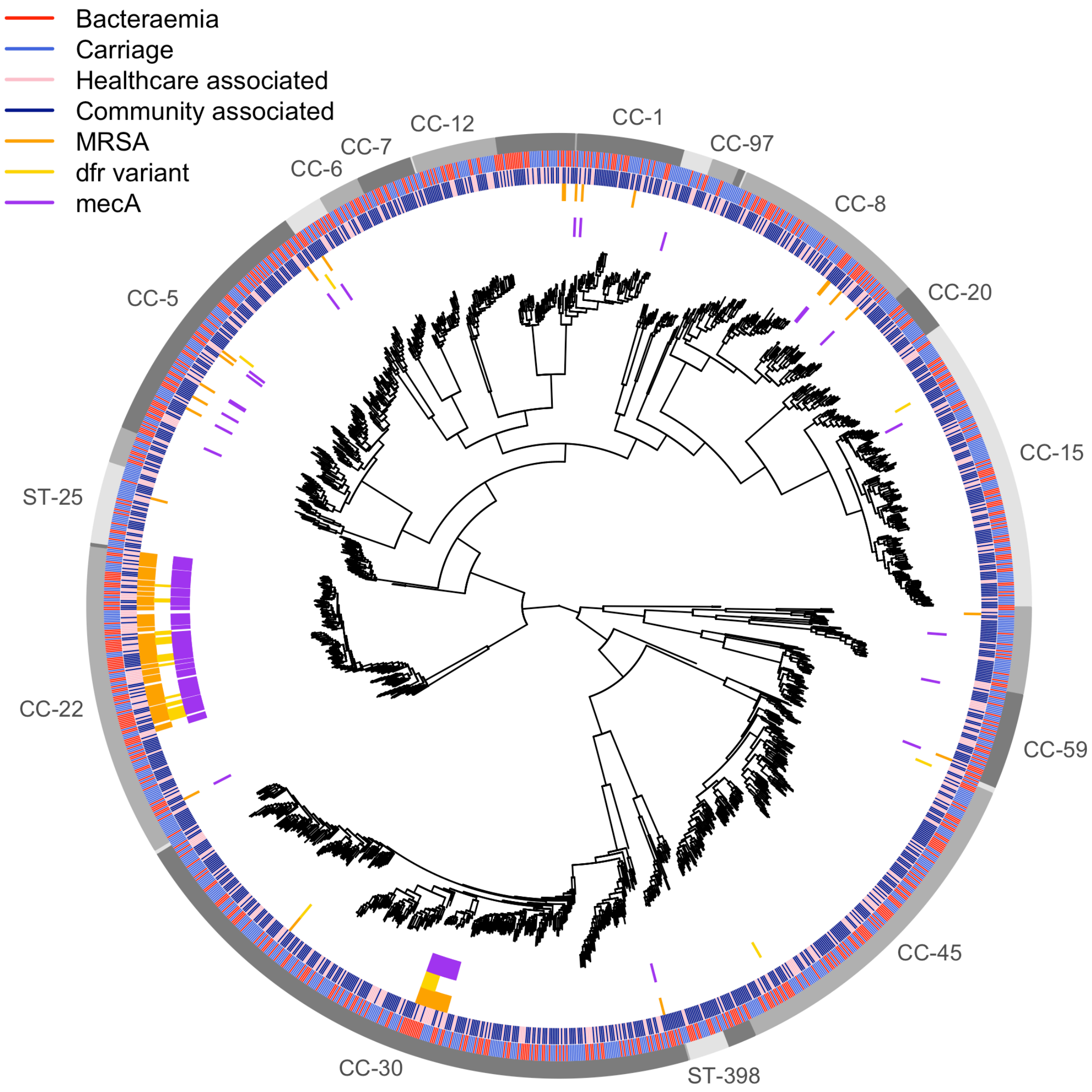

Figure S6 Presence of kmers that are significantly associated with bacteraemia-vs-carriage among 1610 isolates.

Branch lengths have been square-root transformed to better discriminate closely related lineages. The outer ring indicates clusters with a shared lineage named by the clonal complex (or ST if only a single ST was in the cluster). The second outermost ring indicates isolate source (blue carriage, red bacteraemia). The third outermost ring indicates whether each isolate was community or healthcare associated. The fourth ring indicates whether an isolate was phenotypically methicillin resistant (orange). The fifth ring marks the presence of kmers that encode a trimethoprim conferring variant in *dfrB* (yellow). The innermost ring indicates the presence of kmers mapping to the sequence of *mecA* (SAR0039).

A

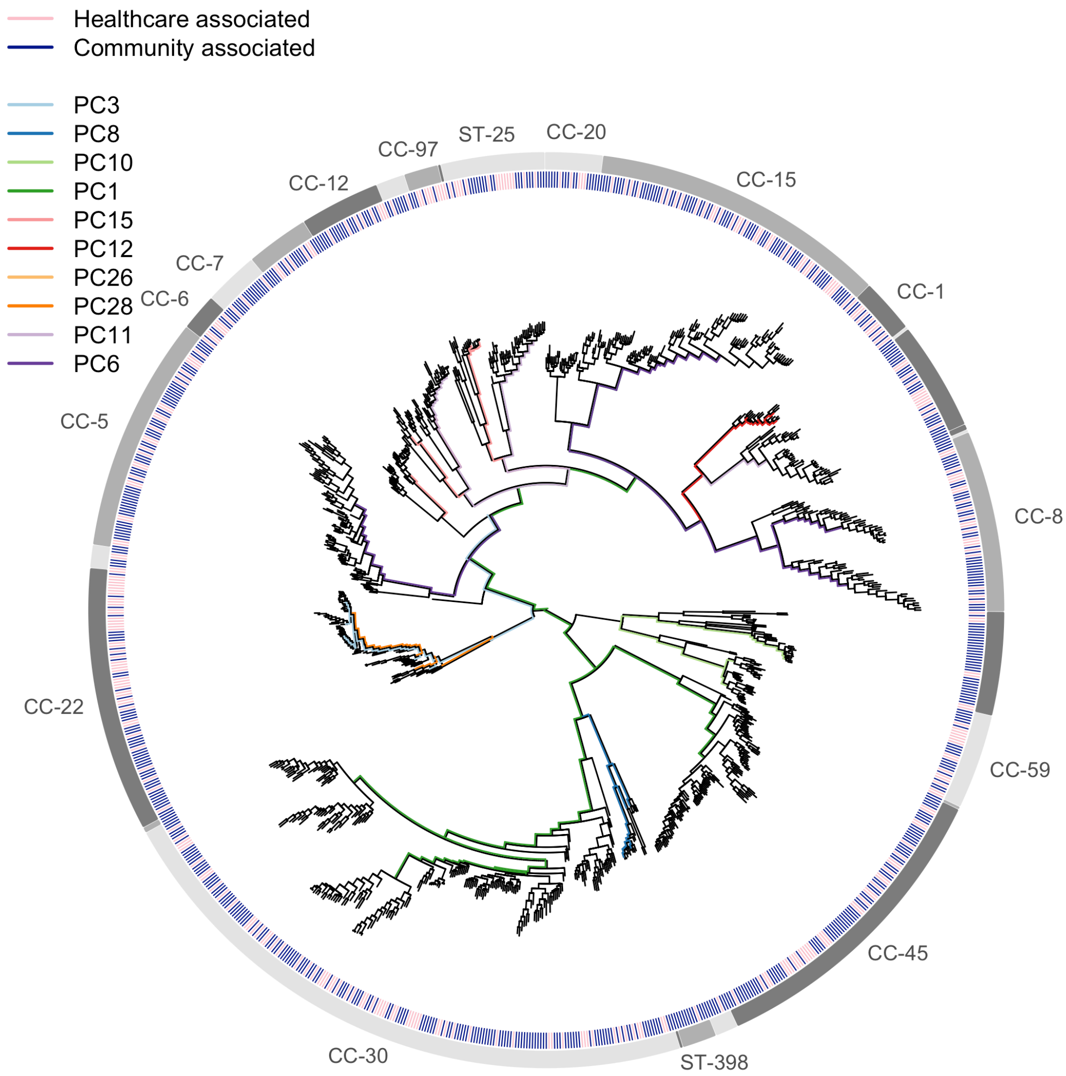
Healthcare associated
Community associated
PC3
PC8
PC10
PC1
PC15
PC12
PC26
PC28
PC11
PC6
CC-97
ST-25
CC-20
CC-15
CC-12
CC-7
CC-6
CC-1
CC-5
CC-8
CC-22
CC-59
CC-45
CC-30
ST-398

B

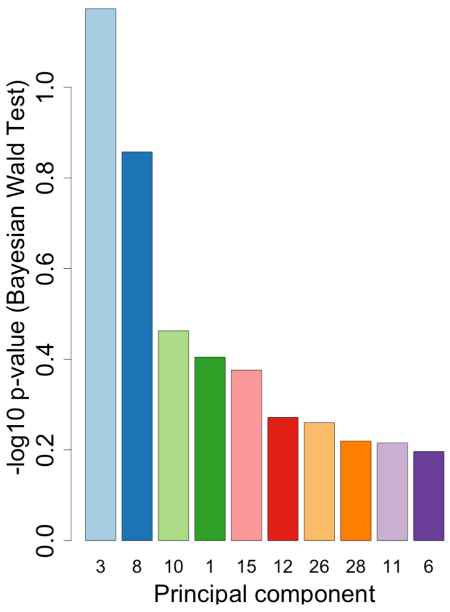
-log10 p-value (Bayesian Wald Test)
0.0
0.2
0.4
0.6
0.8
1.0
3
8
10
1
15
12
26
28
11
6
Principal component

Figure S7: Association of Principal Components with HA-vs-CA carriage. (A) The branches corresponding to the 10 lineages most significantly associated with HA-vs-CA carriage are coloured on a maximum likelihood phylogeny of the study isolates. Branch lengths have been square-root transformed to discriminate closely related lineages. Clonal complexes are denoted in the outermost ring. The second outermost ring indicates isolate source (blue community, pink healthcare) **(B)** Significance (negative $\log_{10}$ *p*-values) of the 10 most significantly associated PCs. A Bonferroni corrected threshold for significance is $10^{-4.3}$.

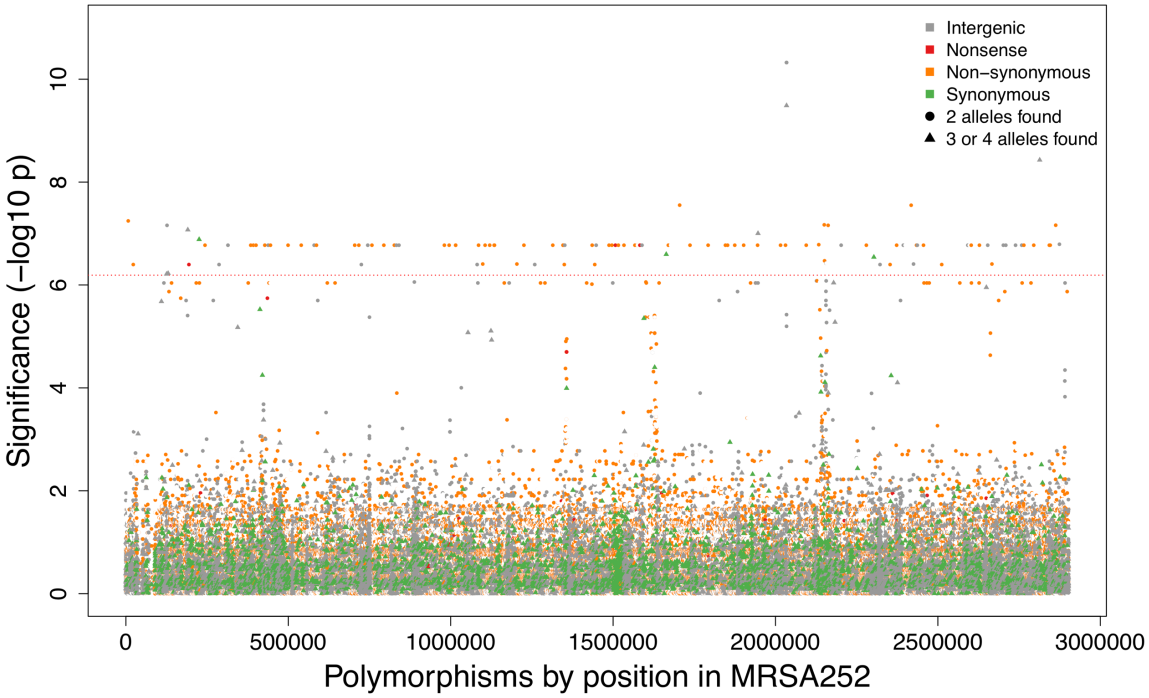

Figure S8: Manhattan plot of SNP associations with HA-vs-CA *S. aureus* carriage. The significance of each SNP ($-\log_{10}$ *p* value) is plotted according to location on the 2.9MB MRSA252 reference genome, with control for population structure. SNPs are coloured according to their predicted effect on protein, and shaped according to the number of alleles found at that site in the study set. We applied Bonferroni correction to the intended 5% false positive rate given the number of SNP phylopatterns to obtain a genome-wide significance threshold of $10^{-6.2}$ (red dotted line).

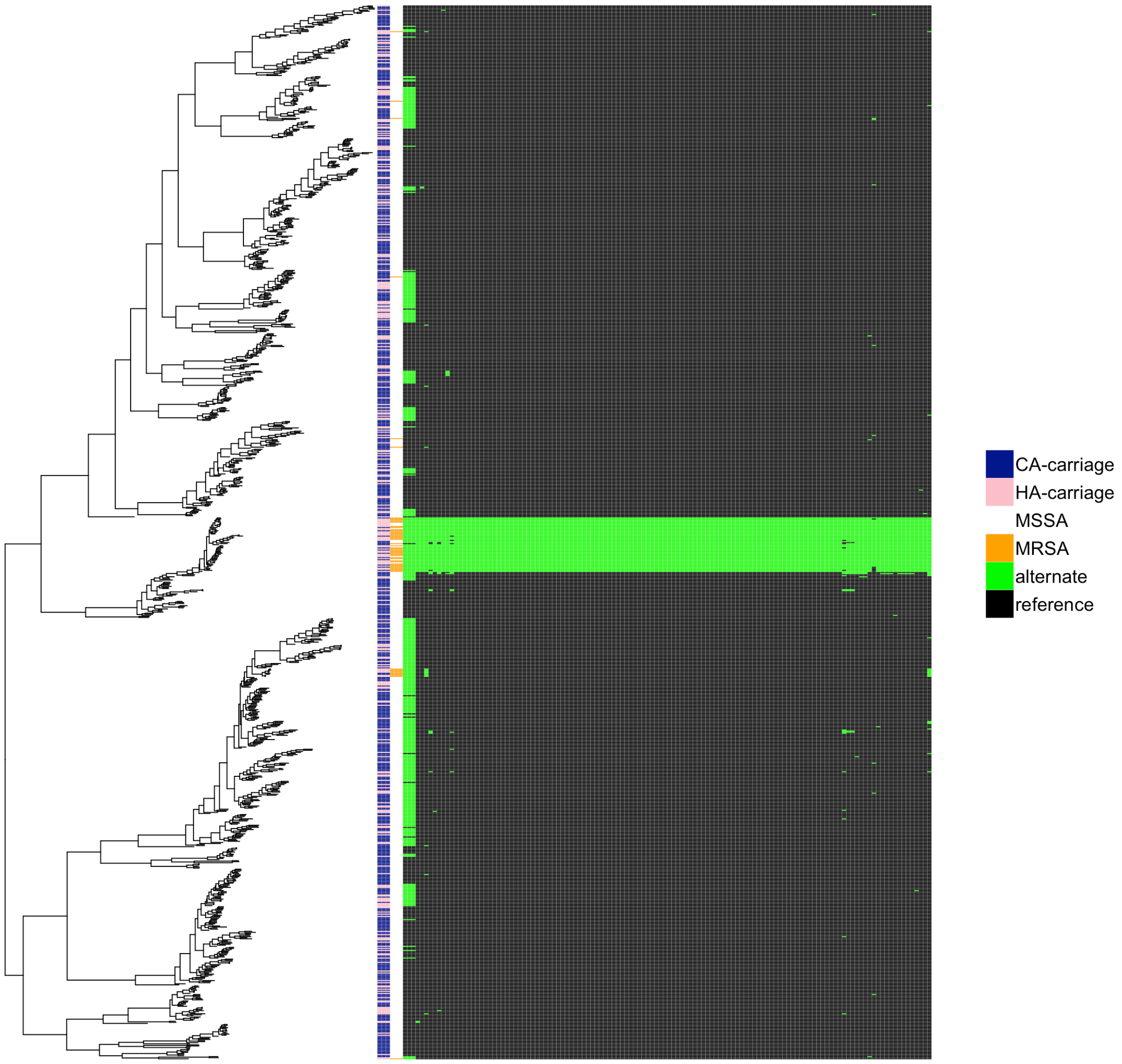

Figure S9: Predicted presence or absence of SNPs significantly associated with HA-vs-CA carriage among the carriage population. A maximum likelihood phylogeny of 984 carriage isolates is plotted with a matrix representing phenotypes concatenated across the most significant SNP genotypes, in order of the significance of association. A maximum likelihood tree of carriage isolates is annotated with the predicted origin of carriage (pink HA, blue CA), the presence of MRSA (orange). The remaining columns represent the presence of the reference (black) or alternate (green) allele for all significant SNPS (after imputation) which were biallelic in the study population.

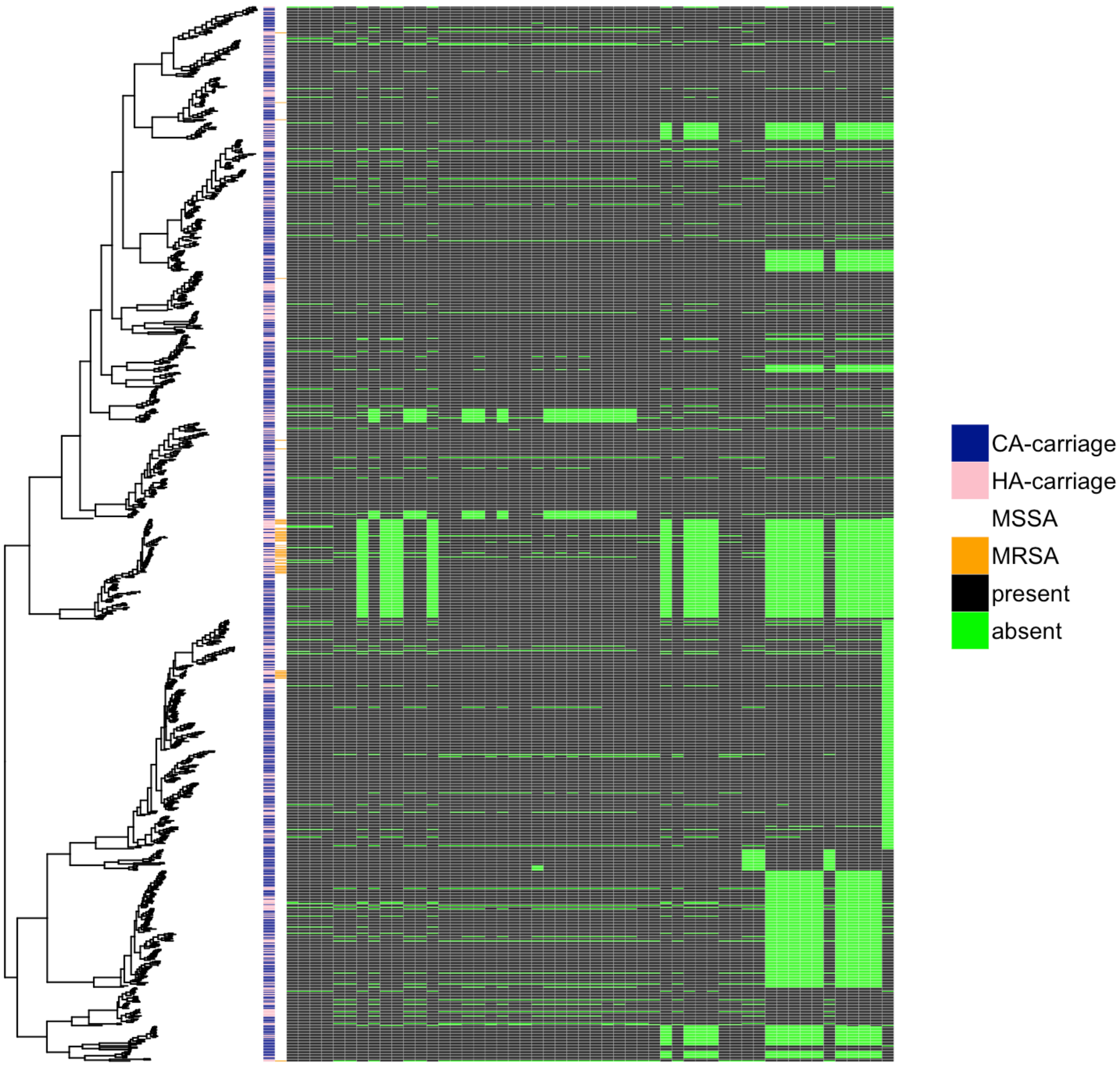

Figure S10: Presence of significant kmers in the population of CA and HA carriage. A maximum likelihood tree of carriage isolates is annotated with the predicted origin of carriage (pink HA, blue CA), the presence of MRSA (orange) and the presence (black) or absence (green) of each pattern of kmers mapping to *prsA*, in decreasing order of statistical significance left to right.

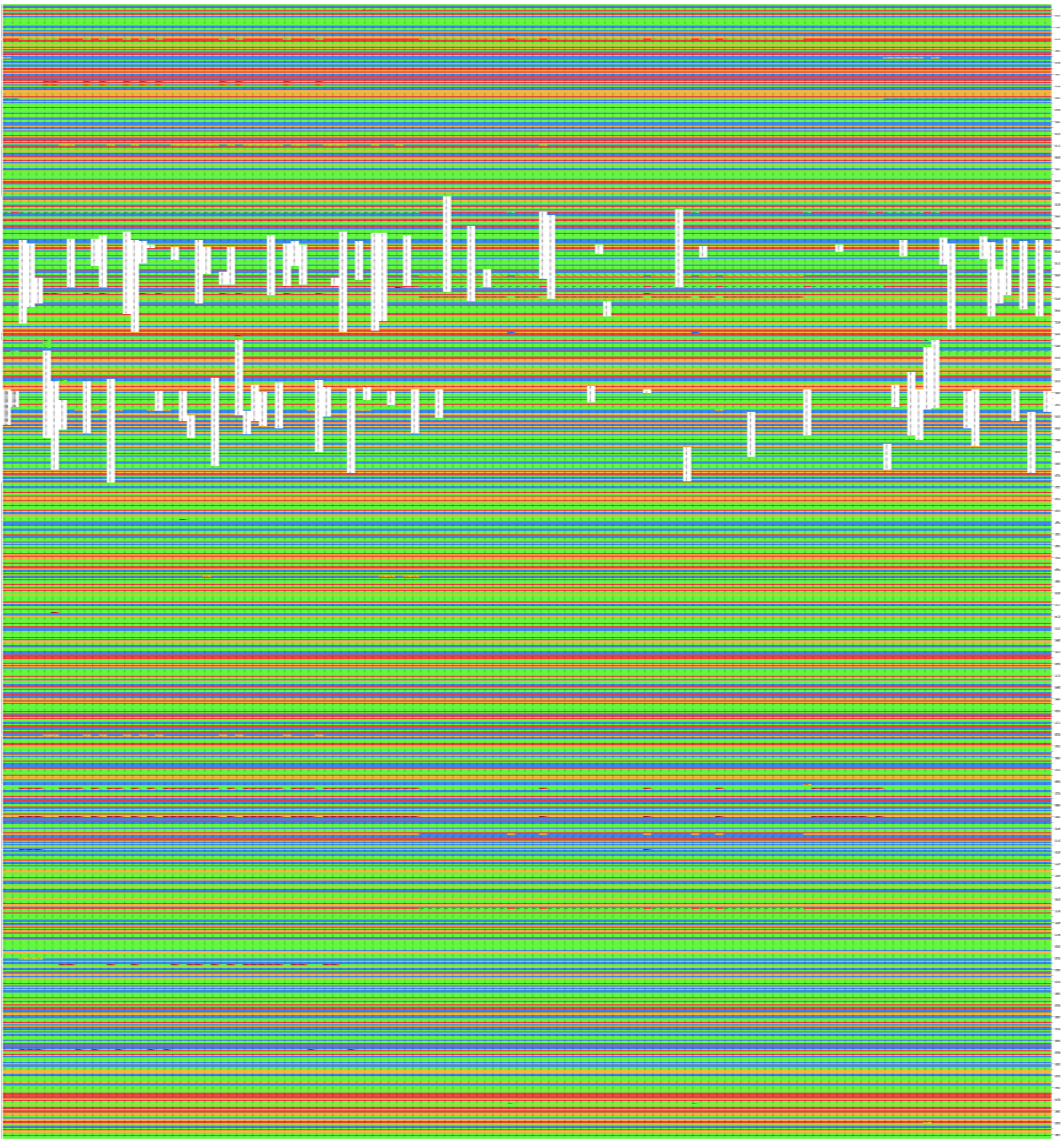

Figure S11: Predicted sequence of *prsA* in 131 HA-carriage genomes. The predicted sequences from 131 HA carriage genomes with the lowest number of significant *prsA* kmers found were aligned against the reference (NC_002952.2:2011797-2012759), and are displayed here aligned vertically and coloured by nucleotide (A green, C orange, G red, T blue, null – white) A plurality of variation is found, including SNPs and short deletions up to 87bp. Deletions are found across bases 164-406; the absence of kmers mapping to bases 177-394 was associated with HA carriage (Figure 3B).

**Focus**	***n***	**%**	**UKCIRG (%)**
Central venous catheter	101	10.9	18.2
Peripheral venous catheter	47	5.1	5.8
Endocarditis	42	4.7	5.8
Soft tissue	244	26.4	19.5
Respiratory	52	5.6	3.7
Urinary	35	3.8	(NA)
Thrombus	11	1.2	(NA)
Vascular implant	17	1.8	(NA)
Other	134	14.5	13.6
Not established	235	25.5	18.8
(Missing data)	94	9.2%	

Table S1: Focus of infection for cases (n=1017) of *Staphylococcus aureus* bacteraemia included, and compared with focus of infection reported in a large cohort of SAB in the UK.[6]

Centre	All cause mortality by 7 days	All cause mortality by 30 days	All cause mortality by 90 days	Morality data missing
Oxford	94	180	228	6/670 (0.9)
N=664	(14.1)	(27.1)	(34.3)	
Brighton	15	27	38	70/187 (37.4)
N=117	(12.8)	(23.1)	(32.5)	
Plymouth	14	38	54	6/160 (3.7)
N=154	(9.1)	(24.7)	(35.1)	
**All**	123	245	320	92/1017 (9.0)
**N = 925**	(13.3)	(26.5)	(34.6)	

Table S2: Mortality at 7, 30 and 90 days, total (percentage). Differences between centres and 7, 30 and 90 days were not statistically significant (p=0.25, p=0.6 and p=0.9, χ^2 test with 2 degrees of freedom)

Table S3: SNPs showing significant association with HA-vs-CA carriage

Table S4: Kmers showing significant association with HA-vs-CA carriage

Table S5: Summary of data availability for all strains included in study, including data deposition in SRA
